# Unanchored ubiquitin chains do not lead to marked alterations in gene expression in *Drosophila melanogaster*

**DOI:** 10.1242/bio.043372

**Published:** 2019-05-16

**Authors:** Jessica R. Blount, Danielle N. Meyer, Camille Akemann, Sean L. Johnson, Katherine Gurdziel, Tracie R. Baker, Sokol V. Todi

**Affiliations:** 1 Department of Pharmacology, Wayne State University School of Medicine, Detroit, MI 48201, USA; 2 Department of Obstetrics and Gynecology, Wayne State University School of Medicine, Detroit, MI 48201, USA; 3 Institute of Environmental Health Sciences, Wayne State University, Detroit, MI 48201, USA; 4 Department of Neurology, Wayne State University School of Medicine, Detroit, MI 48201, USA

**Keywords:** DAVID, *Drosophila melanogaster*, Pathway Analysis, Proteolysis, RNA-Seq, Ubiquitin

## Abstract

The small protein modifier ubiquitin regulates various aspects of cellular biology through its chemical conjugation onto proteins. Ubiquitination of proteins presents itself in numerous iterations, from a single mono-ubiquitination event to chains of poly-ubiquitin. Ubiquitin chains can be attached onto other proteins or can exist as unanchored species, i.e. free from another protein. Unanchored ubiquitin chains are thought to be deleterious to the cell and rapidly disassembled into mono-ubiquitin. We recently examined the toxicity and utilization of unanchored poly-ubiquitin in *Drosophila melanogaster*. We found that free poly-ubiquitin species are largely innocuous to flies and that free poly-ubiquitin can be controlled by being degraded by the proteasome or by being conjugated onto another protein as a single unit. Here, to explore whether an organismal defense is mounted against unanchored chains, we conducted RNA-Seq analyses to examine the transcriptomic impact of free poly-ubiquitin in the fly. We found ∼90 transcripts whose expression is altered in the presence of different types of unanchored poly-ubiquitin. The set of genes identified was essentially devoid of ubiquitin-, proteasome-, or autophagy-related components. The seeming absence of a large and multipronged response to unanchored poly-ubiquitin supports the conclusion that these species need not be toxic *in vivo* and underscores the need to re-examine the role of free ubiquitin chains in the cell.

## Abstract (precis)

**Summary:** Our *Drosophila* studies indicate the lack of a marked, coordinated response towards unanchored poly-ubiquitin in flies, suggesting that untethered ubiquitin chains are not necessarily problematic in intact organisms.

## INTRODUCTION

Cellular and organismal physiology and homeostasis are regulated at multiple, inter-dependent levels that extend from DNA-based regulation of gene expression to the epigenetic control of genes themselves and of their products. Among the more flexible systems of epigenetic control is the post-translational modification of cellular proteins by various adducts, including ubiquitination, phosphorylation, methylation and acetylation. Ubiquitination represents a highly malleable system of post-translational regulation of proteins and of the complexes in which they participate (Komander and Rape, 2012; Swatek and Komander, 2016). Ubiquitin (Ub), itself a small protein of approximately 8.5 kDa, is highly conserved among all eukaryotic species and regulates proteins in various ways, from tagging them for proteasomal degradation to directing their participation in cellular signaling pathways (Komander and Rape, 2012; Ristic et al., 2014; Asaoka et al., 2016).

**Fig. 1.**
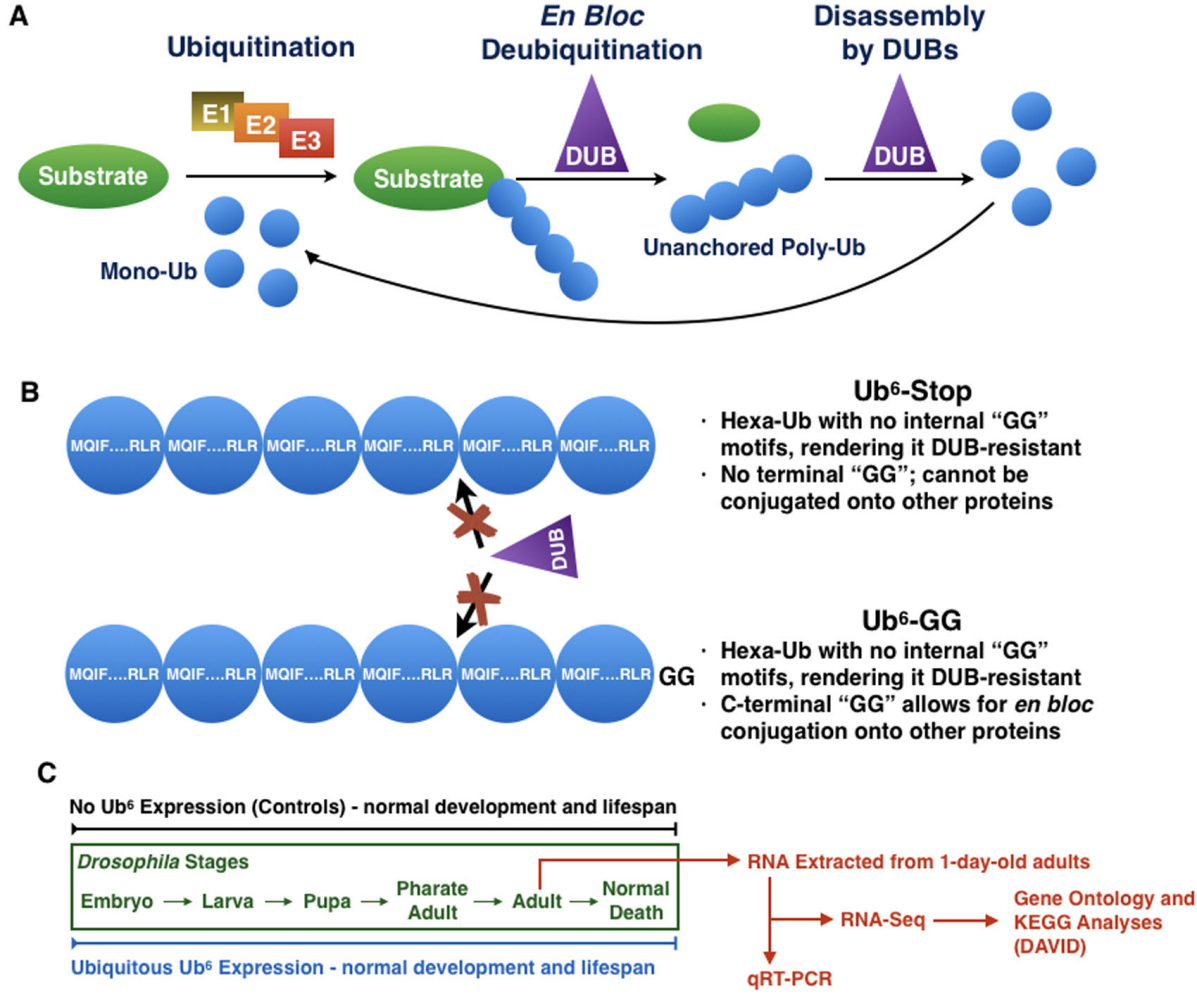
**Unanchored poly-Ub.** (A) Unanchored poly-Ub can arise after E1/E2/E3 cycles build a Ub chain onto a substrate, after which a DUB removes the chain as a single species. It is believed that these untethered chains are then dismantled by additional DUBs to yield mono-Ub that can be recycled in new ubiquitination events. (B) Schematic of the two types of Ub^6^ chains we designed for expression in *Drosophila*. Both Ub^6^-Stop and Ub^6^-GG are head-to-tail hexa-Ub that cannot be dismantled by DUBs. Ub^6^-GG, but not Ub^6^-Stop, can be conjugated onto other proteins. (C) Ubiquitous Ub^6^ expression does not affect the development or the lifespan of the fly (Blount et al., 2018). One-day-old adults were collected and processed for RNA-Seq and qRT-PCR analyses.

Ub conjugation onto another protein – what is termed ubiquitination – requires the concerted effort of three types of enzymes: an E1 activating enzyme, an E2 conjugating enzyme and an E3 ligase (Fig. 1A). This ATP-dependent process results in an iso-peptide bond between the C-terminal ‘GG’ motif of a Ub molecule and the substrate protein, typically at a lysine residue. Ub itself can also become ubiquitinated, resulting in a poly-Ub chain defined by the specific modified lysine residue or, in the case of M1/linear chains, the methionine residue of Ub (Komander and Rape, 2012; Yau and Rape, 2016; Yau et al., 2017). The type of chain created has a distinct effect on the fate of its substrate protein; for instance, K48 chains are known for their involvement in targeting proteins for proteasomal degradation (Thrower et al., 2000). Ubiquitination is a reversible process; deubiquitinases (DUBs) remove Ub from a protein or edit the length and type of a Ub chain (Fig. 1A; Swatek and Komander, 2016).

Unanchored Ub chains – that is, poly-Ub that is not tethered onto a substrate protein – also exist in the cell. Unanchored Ub chains can arise when a DUB removes an intact chain from a protein, or they can be generated anew through E1/E2/E3 cycles. Although unanchored poly-Ub is not well understood, it has been implicated as a participant in several cellular processes, including NF-κB signaling and autophagy (Swatek and Komander, 2016; Clague et al., 2013; Emmerich et al., 2013; Hao et al., 2013; Reyes-Turcu et al., 2008, 2009; Reyes-Turcu and Wilkinson, 2009; Braten et al., 2012; Keusekotten et al., 2013; Elliott and Komander, 2016; Lee et al., 2016). The prevailing view is that unanchored Ub chains are quickly disassembled by DUBs and recycled as mono-Ub (Fig. 1A) (Komander and Rape, 2012; Clague et al., 2013; Ristic et al., 2014; Komander et al., 2009). Studies in yeast and in cultured mammalian cells have suggested that the buildup of free poly-Ub might become toxic by, for example, perturbing Ub-dependent proteasomal degradation (Piotrowski et al., 1997; Doelling et al., 2001; Amerik and Hochstrasser, 2004; Wang et al., 2014; Amerik et al., 1997; Dayal et al., 2009).

Intriguingly, when we examined the toxicity of untethered chains *in vivo* we observed that the presence of free Ub chains is not necessarily deleterious to an intact organism, *Drosophila melanogaster* (Table S1; Blount et al., 2018). For these studies in *Drosophila*, we designed head-to-tail hexa-Ub chains that lack ‘GG’ motifs (Ub^6^; Fig. 1B), making them resistant to cleavage by DUBs; these chains resemble linear, unanchored Ub chains that are endogenously present (Komander and Rape, 2012; Clague et al., 2013; Ristic et al., 2014; Komander et al., 2009). We observed that when expressed at high levels in all fly tissues and at all developmental and adult stages, unanchored poly-Ub does not negatively impact the lifespan of the fly (Table S1; Blount et al., 2018). It has been suggested that free poly-Ub could interfere with the proteasome. However, we observed no deficiencies in proteasome subunit expression or function in intact flies; in fact, untethered poly-Ub were degraded by the fly proteasome (Blount et al., 2018).

Throughout our studies (Blount et al., 2018), we became confident that unanchored poly-Ub is not inherently or especially toxic, but it was still unclear whether their presence induces a concerted cellular response against them. While the Ub^6^ that we constructed resemble linear, unanchored poly-Ub, their inability to be cleaved is unnatural. Does the introduction of these exogenous chains bring about an organismal response, or are they as readily tolerated as they seem to be? Is there an upregulation of dismantling DUBs, like USP5, which is widely reported to disassemble free poly-Ub (Amerik and Hochstrasser, 2004; Reyes-Turcu et al., 2009; Reyes-Turcu and Wilkinson, 2009; Ristic et al., 2016; Komander and Rape, 2012; Komander et al., 2009; Scaglione et al., 2011; García-Caballero et al., 2014)? Is there a change in the expression of E2/E3 complexes that might be able to take advantage of premade chains? To answer some of these questions, for the present study we conducted RNA-Seq analyses, where we observed that ubiquitous expression of Ub^6^ induces significant changes in the expression of approximately 90 fly genes, with no clear indication of a specific cellular response mounted. Our examinations did not reveal a coordinated effect on pathways that are known to involve unanchored poly-Ub. According to these results, unanchored poly-Ub does not elicit a marked organismal response in *Drosophila*, suggesting that these Ub species are not inherently problematic.

## RESULTS

### Unanchored Ub chain design and expression in *Drosophila* for RNA-Seq analyses

As a strategy to study unanchored poly-Ub in *Drosophila*, we designed two types of Ub chain transgenes, each consisting of six Ub in tandem, without internal di-glycine, ‘GG’ motifs that are required for dismantling into mono-Ub by DUBs (Fig. 1B; Blount et al., 2018). The first chain type, Ub^6^-Stop, also lacks the C-terminal ‘GG’ motif required for conjugation onto substrate proteins. The second type, Ub^6^-GG, contains a C-terminal ‘GG’ motif, allowing the full chain to form iso-peptide bonds onto other proteins in mammalian cells and *in vivo* in the fly, as we demonstrated before (Blount et al., 2018). Although the use of these chains introduces exogenous poly-Ub, this strategy permits us to directly examine the effects of intact, free poly-Ub on the *Drosophila* transcriptome. Presently, there is a lack of tools to more directly investigate unanchored poly-Ub in the fly; for example, targeting of DUBs implicated in free Ub chain disassembly would also impact other protein substrates that these DUBs have.

We utilized the binary Gal4-UAS expression system to drive our Ub^6^ transgenes in the fly. In this system, transgenes with upstream activating sequence (UAS) sites are activated under the control of the transcription factor Gal4, itself expressed in the pattern of a specific gene (Brand and Perrimon, 1993; Brand et al., 1994). For our work in this study, we selected the Gal4 driver sqh-Gal4 (Kiehart et al., 2004; Franke et al., 2006, 2010; Todi et al., 2005, 2008) to express either form of Ub^6^ in all fly tissues, throughout development and in adults. This driver employs the promoter and expression pattern of the gene *spaghetti squash* (*sqh*), which encodes the regulatory light chain of non-muscle type 2 myosin. sqh-Gal4 is a strong driver that leads to high levels of UAS-based transgene expression (such as our Ub^6^), during all developmental stages and throughout adulthood. We and others have used this driver in the past with robust outcomes, including lethality during various developmental stages and in adults as a result of the knockdown of various genes, and high toxicity from the expression of mutated or toxic proteins (Ristic et al., 2016; Sutton et al., 2017; Tsou et al., 2015, 2016, 2012; Franke et al., 2006, 2010, 2005; Todi et al., 2005; Costa et al., 2016).

Our previously published work showed that the ubiquitous expression of Ub^6^ (via sqh-Gal4) had no significant effect on the development or lifespan of adult flies under normal conditions or during heat stress (30°C), indicating that robust levels of Ub^6^ are not especially detrimental (Fig. 1C; Table S1; Blount et al., 2018). Still, the possibility remains that cells could mount a response against them. One may surmise that in response to the presence of Ub^6^, DUBs or proteasomal proteins might be upregulated to attempt to clear the chains from the cell. Conceivably, Ub^6^ might also influence normal cellular processes, for instance by its recruitment into pathways that involve unanchored, linear poly-Ub, such as NF-κB signaling (Asaoka et al., 2016; Damgaard et al., 2016; Elliott and Komander, 2016; Emmerich et al., 2013; Keusekotten et al., 2013). Thus, we set out to examine if there are changes at the transcriptome level in response to Ub^6^.

We reasoned that we could detect changes in the fly transcriptome as a result of the expression and presence of untethered poly-Ub through RNA-Seq analysis. We selected to examine adult flies that were one day old as a middle point between developmental stages and adulthood, neither of which was impacted by the expression of Ub^6^ (Table S1; Blount et al., 2018). We extracted total RNA from one-day-old whole flies using TRIzol. The isolated RNA was then quality tested by electrophoretogram, RNA Integrity Number and the ratio of the 28S:18S RNA bands, and RNA-Seq was performed by the Applied Genomics Technology Center at Wayne State University (please see the Materials and Methods). The differentially expressed transcripts were analyzed using the Database for Annotation, Visualization and Integrated Discovery (DAVID) (Huang et al., 2009b).

While Ingenuity Pathway Analysis (IPA) is often the tool of choice to analyze RNA-Seq results, human orthologues exist for only about two-thirds of the transcripts affected by either form of Ub^6^ (Dataset S1) and the IPA databases available to us do not emphasize *Drosophila* genes. Because the success of IPA is heavily dependent on having access to the most applicable database (Huang et al., 2009a), we opted to perform our analyses using DAVID. DAVID avoids stretching our observations to fit within the context of organisms other than *Drosophila*, or excluding *Drosophila*-specific genes involved in pathways of interest. Other fly laboratories have shown that DAVID analysis recognizes and compares genes from the fly genome, while also providing the tools to group functionally related genes and terms into a manageable number of biological modules (Table S2; Crona et al., 2015; Huang et al., 2007, 2009b; Sherman et al., 2007; Gramates et al., 2017). Fig. 1C outlines our experimental workflow. We note at this point that all of the genes discussed here are identified by their *Drosophila* symbol/name. The names of their potential human orthologues, where applicable, are also provided in the text and tables.

### DAVID analyses

**Fig. 2.**
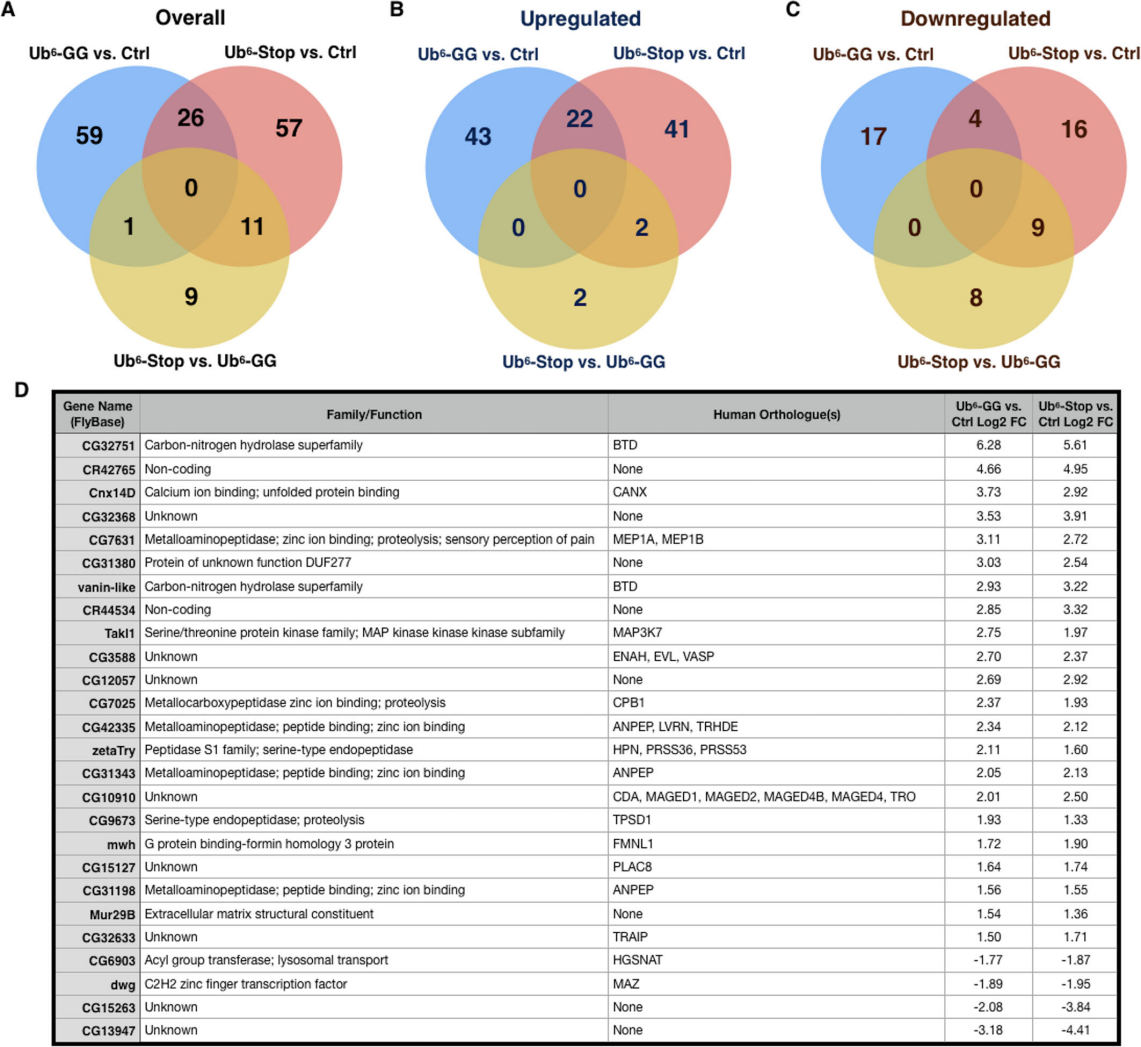
**Venn diagrams (http://bioinformatics.psb.ugent.be/webtools/Venn/) depict overlap in the number of differentially expressed (absolute Log2 fold change >1, FDR<0.05) genes as determined by RNA-Seq at each level of comparison (Ub^6^-GG versus control, Ub^6^-Stop versus control, Ub^6^-Stop versus Ub^6^-GG).** (A) All genes, (B) upregulated genes only, (C) downregulated genes only. (D) Overlapping, differentially-expressed transcripts that are consistent in direction in both Ub^6^-GG versus control and Ub^6^-Stop versus control comparisons. FC, fold change. All genes are identified by their *Drosophila* symbol/name, with ‘CG’ denoting that the gene has not yet been named in *Drosophila*. Transcripts were researched using Flybase.org and any information on function and human orthologues is displayed in the table.

We found a limited number of altered transcripts in flies expressing non-conjugatable and conjugatable untethered Ub^6^ chains: 94 transcripts were altered in Ub^6^-Stop and 86 were altered in Ub^6^-GG flies compared to controls, including 26 transcripts affected in both lines (Fig. 2; Dataset S1). Controls were flies with the same genetic background used to generate Ub^6^ flies, crossed to the sqh-Gal4 driver, ensuring that flies were as comparable as possible at the genetic level. The majority of altered transcripts was upregulated (65 from each condition), with 22 transcripts overlapping between groups (Fig. 2). Only 30% of all identified *Drosophila* transcripts have assigned gene names, indicating that most of the affected genes have not drawn sufficient genetic or functional attention in the fly, and 27% of the genes have no predicted function.

To place these altered transcripts into physiological context, we submitted the differentially expressed transcripts to DAVID (Huang et al., 2009b). This allowed us to determine enriched gene ontology terms and pathways, assessing biological processes, molecular functions and KEGG (Kyoto Encyclopedia of Genes and Genomes) pathways (Tables 1–3). Five biological process terms (proteolysis, peptide catabolic process, mannose metabolic process, protein deglycosylation and melanin biosynthetic process from tyrosine) were enriched in upregulated Ub^6^-GG transcripts, whereas for Ub^6^-Stop, three terms (proteolysis, peptide catabolic process and transmembrane transport) were enriched in upregulated and six terms (carbohydrate metabolic process, folic acid-containing compound biosynthesis process, tetrahydrofolate interconversion, *de novo* IMP biosynthetic process, one-carbon metabolic process and oxidation-reduction process) were enriched in downregulated transcripts. The biological process terms ‘proteolysis’ and ‘peptide catabolic process’ were enriched in upregulated transcripts from both groups, the former associated with ∼22% of all upregulated Ub^6^-GG transcripts and ∼18% of upregulated Ub^6^-Stop transcripts (Table 1; Table S3 lists all genes for each term). Five molecular function terms, the most prominent of which were ‘serine-type endopeptidase activity’ and ‘peptide binding’ were enriched in upregulated transcripts from both groups. The molecular function category overall reported nine enriched terms in upregulated Ub^6^-GG transcripts, as well as five upregulated and six downregulated transcripts for Ub^6^-Stop (Table 2; Table S4 lists all genes associated with each term).

**Table 1.**
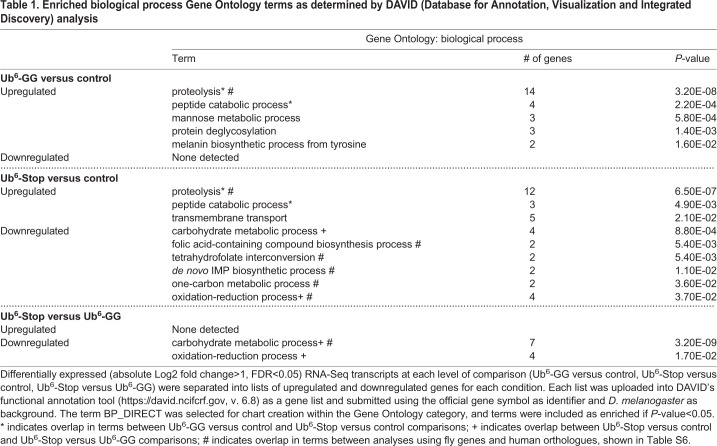
**Enriched biological process Gene Ontology terms as determined by DAVID (Database for Annotation, Visualization and Integrated Discovery) analysis**

**Table 2.**
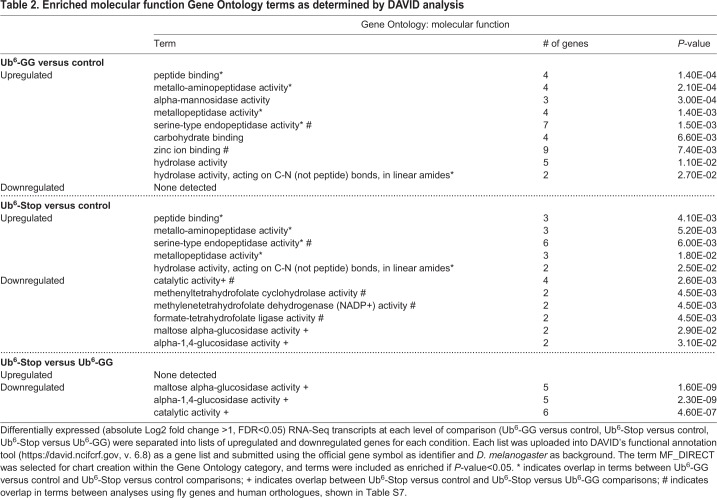
**Enriched molecular function Gene Ontology terms as determined by DAVID analysis**

**Table 3.**
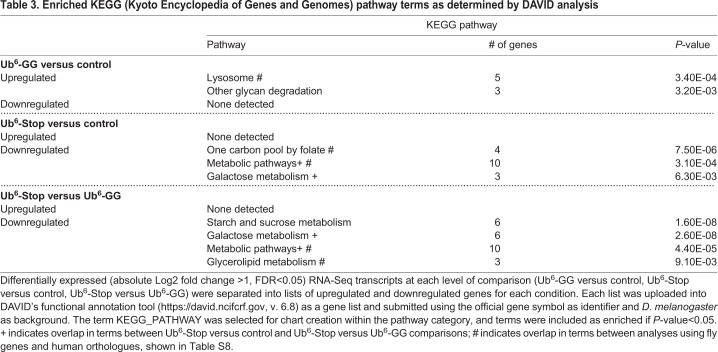
**Enriched KEGG (Kyoto Encyclopedia of Genes and Genomes) pathway terms as determined by DAVID analysis**

As in other transcriptomic studies (Gajan et al., 2016; Kučerová et al., 2016), we assessed differentially expressed genes for both enriched Gene Ontology terms and KEGG pathways, in order to provide both a gene-specific and broader pathway context. For KEGG analysis, two pathways (lysosome and other glycan degradation) were enriched in upregulated Ub^6^-GG transcripts, with three (one carbon pool by folate, metabolic pathways and galactose metabolism) enriched in downregulated Ub^6^-Stop transcripts. Although few KEGG pathways were enriched, the term ‘metabolic pathways’ was associated with 34% of downregulated transcripts in Ub^6^-Stop flies (Table 3; Table S5 lists all genes associated with each term). The relatively modest number of enriched gene ontology terms and pathways is not unexpected, considering the limited number of differentially expressed transcripts that reached statistical significance.

Next, we examined the possibility that unanchored Ub chains that cannot be conjugated elicit a response different from chains that can be conjugated. Thus, we directly compared flies expressing Ub^6^-Stop to those expressing Ub^6^-GG. Only 21 transcripts were altered in Ub^6^-Stop compared to Ub^6^-GG, 17 of which were downregulated (Fig. 2 and Dataset S1; no transcripts were altered across all comparisons). We again relied on DAVID to analyze differentially expressed transcripts for gene ontology terms and pathways (Tables 1–3; Tables S3–S5 list all genes associated with each term). Two biological processes (carbohydrate metabolic process and oxidation-reduction process) and three molecular functions (maltose alpha-glucosidase activity, alpha-1,4-glucosidase activity and catalytic activity) were enriched in downregulated Ub^6^-Stop transcripts in comparison to both Ub^6^-GG and controls (Tables 1,2; Tables S3,S4). For KEGG analysis, four pathways were enriched in downregulated Ub^6^-Stop transcripts, with two of the four (galactose metabolism and metabolic pathways) enriched in comparison with both other conditions (Table 3; Table S5). While these transcriptomic and pathway analysis outcomes suggest a response specific to flies expressing non-conjugatable free Ub chains, the small number of differentially expressed transcripts limits this interpretation.

Lastly, we used DAVID to analyze only the set of human genes that have fly orthologues, in case additional or different pathways arise that might not have been captured by analyzing the fly genes exclusively. As shown in Tables S6–S8, there was general agreement with the fly-based DAVID analyses. The biological processes, molecular functions and pathways that were represented by the largest numbers of fly genes were well conserved between the two sets of analyses, including proteolysis and carbohydrate metabolic process, zinc-ion binding and serine-type endopeptidase activity, and lysosome and metabolic pathways. Still, biological processes and molecular functions arose that were not observed from fly-based gene analysis. These differences were most often represented by small numbers of genes, generally two to four. Among biological processes, these include upregulation of regulation of cell shape, response to pH and termination of signal transduction (comparing Ub^6^-GG versus control), upregulation of biotin metabolism (comparing Ub^6^-Stop versus control), and downregulation of amino acid transport and protein tetramerization (comparing Ub^6^-Stop versus control). Among molecular functions that emerged from the human gene-based analyses, apolipoprotein binding and mannose binding were upregulated (comparing Ub^6^-GG versus control), zinc-ion binding was upregulated (comparing Ub^6^-Stop versus control), and electron carrier activity and oxidoreductase activity were downregulated (comparing Ub^6^-Stop versus Ub^6^-GG). Based on KEGG pathway analyses, the following differences were observed in the human-based analyses when compared to the fly-based analyses: upregulation of metabolic pathways (comparing Ub^6^-GG versus control), downregulation of the biosynthesis of antibiotics and glycerolipid metabolism (comparing Ub^6^-Stop versus control), and downregulation of the biosynthesis of antibiotics (comparing Ub^6^-Stop versus Ub^6^-GG). Collectively, while there are some variations between the two sets of analyses, the overall outcomes are not markedly different. Importantly, considering the small numbers of genes representing the divergences between the two sets of analyses, it is warranted that differences in outcomes be interpreted with caution.

Overall, expression of unanchored Ub chains in *Drosophila* has a seemingly minimal impact on transcriptomic response, as the number of altered transcripts (<100 for each group) is markedly low in comparison to the majority of reports by other, whole-fly RNA-Seq studies (Moskalev et al., 2014; Castillo et al., 2015; MacMillan et al., 2016; Jiang et al., 2017). Although one study reported a comparable 57 genes affected by formaldehyde exposure (Moskalev et al., 2014), most were within the range of hundreds to several thousand genes, in studies ranging from cold acclimation to infection (Moskalev et al., 2014; MacMillan et al., 2016; Jiang et al., 2017; Castillo et al., 2015).

### Validation by qRT-PCR

**Fig. 3.**
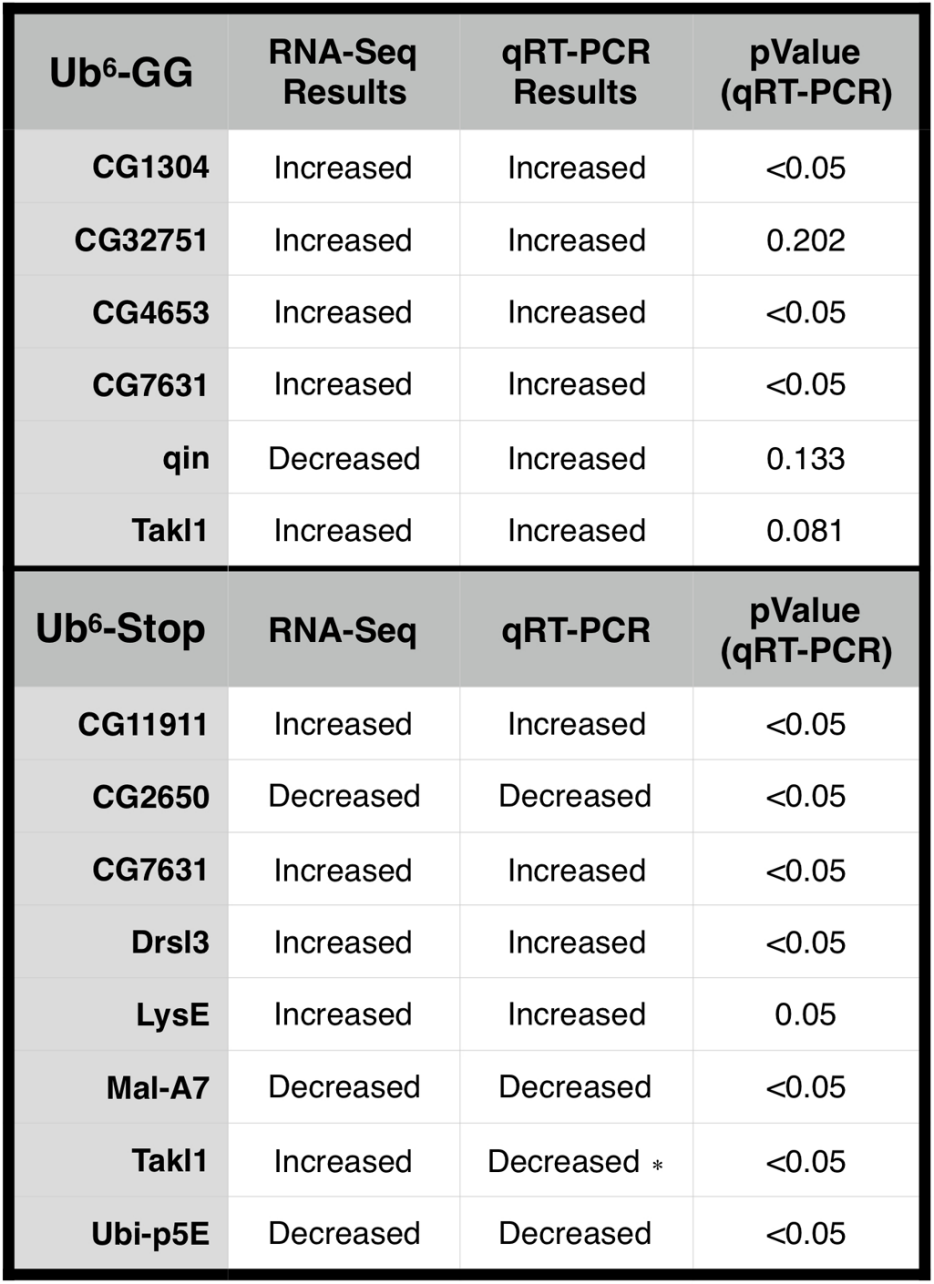
**qRT-PCR validation of differential expression trends for select RNA-Seq hits.** Asterisk indicates inconsistency between RNA-Seq and qRT-PCR results. All *P*-values were determined using a one-tailed Student's *t*-test comparing gene expression fold change.

To validate RNA-Seq observations, we selected twelve genes from several pathways for confirmation by qRT-PCR. cDNA libraries were obtained from the same RNA used for RNA-Seq, as well as from RNA extracted from new genetic crosses, for at least three biological replicates per genotype. All primer sequences are listed in Table S9. In most cases, results from RNA-Seq were confirmed by statistically significant changes, in the same direction, by qRT-PCR (Fig. 3). Our overall confirmation success is well within the range of confirmation reported widely in the literature (Rajkumar et al., 2015; Aanes et al., 2014; Kaur et al., 2012; van Blitterswijk et al., 2013). qRT-PCR-validated transcripts include several genes with marked expression changes observed by RNA-Seq: *CG32751* (∼78-fold increase); *Drsl3* and *LysE* (∼tenfold increase); *Mal-A7* and *CG2650* (∼tenfold decrease) (Dataset S1 lists all log2 fold changes determined by RNA-Seq).

The direction of change for the transcripts that were confirmed to reach statistical significance by qRT-PCR was the same between RNA-Seq and qRT-PCR results, with one exception: *Takl1* in response to Ub^6^-Stop (Fig. 3; please see the Discussion for additional information on Takl1 protein). By RNA-Seq, *Takl1* on the Ub^6^-Stop background nearly missed the FDR cutoff (FDR=0.047; GEO data available online). We interpret this divergence in outcomes for *Takl1* as an indicator of lack of overall change in its expression in the presence of Ub^6^-Stop. It is not uncommon for the direction of change in RNA-Seq results to differ from the direction of change observed from qRT-PCR data (Rajkumar et al., 2015; Aanes et al., 2014; Kaur et al., 2012; van Blitterswijk et al., 2013). These discrepancies can arise for a variety of reasons, including the housekeeping gene used (Kaur et al., 2012) as well as the length of the identified genes (Bullard et al., 2010). The rest of the genes we tested by qRT-PCR, whose differential expression reached statistical significance, matched the direction of fold change observed with RNA-Seq. The expression pattern of the genes assessed by qRT-PCR in the fly is summarized in Table S9, although we note that this table only lists the tissues in which the genes are more highly expressed; they may also be present in other tissues.

## DISCUSSION

Here, we evaluated whether there is an organismal response at the gene expression level in the presence of unanchored poly-Ub in *Drosophila*. Unanchored Ub chains are thought to be transient, toxic residents of the cellular milieu (Piotrowski et al., 1997; Doelling et al., 2001; Amerik and Hochstrasser, 2004; Wang et al., 2014; Amerik et al., 1997; Dayal et al., 2009). However, our previously published work showed that this might not need to be the case *in vivo*: we observed little to no toxicity from the presence of unanchored poly-Ub in intact flies at all ages and stages examined (Blount et al., 2018). Thus, we wondered whether lack of clear toxicity is due to a mounted defense or response, which we elected to start examining by RNA-Seq.

**Fig. 4.**
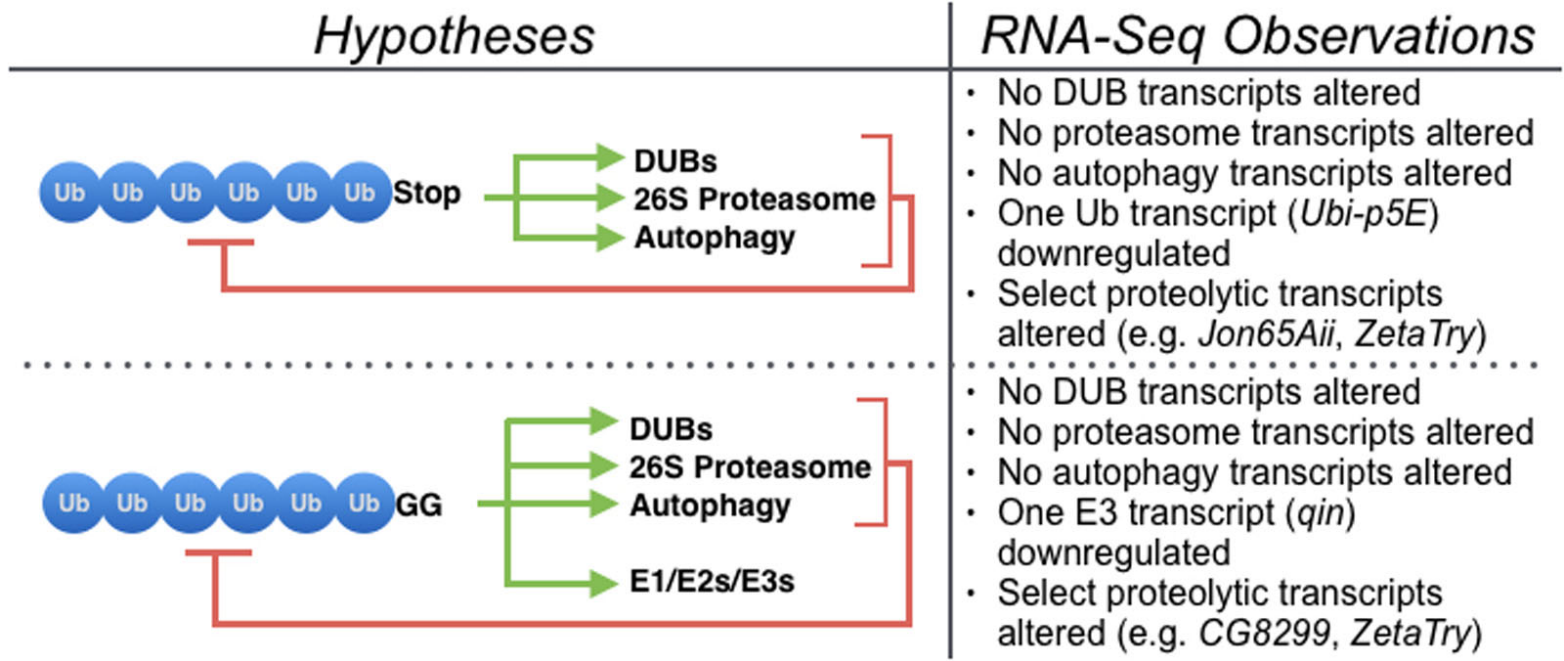
**Overview of the types of genes and pathways that we hypothesized might have been upregulated to dismantle, clear, or re-utilize Ub^6^, compared to the observed outcome by RNA-Seq.**

To model unanchored poly-Ub in the fly, we utilized transgenes that express six Ub moieties in tandem, lacking internal motifs that enable their disassembly into mono-Ub; one transgene encodes Ub^6^ that cannot be attached onto other proteins, whereas the other enables this option (Fig. 1B). Transgenes were expressed in all fly tissues and at all stages of development and in adults, and RNA-Seq analyses were conducted using one-day-old flies. While planning this study, we had several hypotheses on the types of genes that could be affected by untethered Ub chains that we generated and expressed in *Drosophila*. Primarily, we thought that genes involved in Ub-dependent processes and pathways, as well as proteasome-related genes, might be altered. These hypotheses were based on the current notion that unanchored poly-Ub is rapidly disassembled (Komander and Rape, 2012; Clague et al., 2013; Ristic et al., 2014; Komander et al., 2009) and on our previous work showing that Ub^6^ is degraded by the proteasome (Blount et al., 2018). For Ub^6^-GG, we further hypothesized a potential upregulation of Ub conjugation systems that could transfer these chains onto other proteins as single units. These hypotheses went unsupported by our RNA-Seq data; our analyses did not reveal a detectable difference in the transcription of those genes, with the exception of one E3 ligase, *qin* (Fig. 4), whose change at the transcript level was not confirmed through qRT-PCR. qin is a Tudor domain protein involved in the production of piRNAs that repress transposons in germline cells. It contains a RING domain and two B-Box domains, indicating E3 ligase activity, but its E3 function has not been characterized (Sato et al., 2015; Zhang et al., 2014, 2011). The lack of congruency between RNA-Seq and qRT-PCR results for *qin* argues against a marked effect from unanchored, conjugatable poly-Ub on the transcript levels of this E3 Ub ligase.

Insofar as Ub-related genes are concerned, we noted a reduction in the levels of *Ubi-p5E* in the presence of Ub^6^-Stop (Figs 3,4; Dataset S1). *Ubi-p5E* is one of five *Drosophila* genes that encode mono-Ub either as a linear chain of multiple Ub moieties or in fusion to ribosomal components (Lee et al., 1988; Özkaynak et al., 1984; Lund et al., 1985; Redman, 1994; Redman and Rechsteiner, 1989; Baker and Board, 1991). Ub genes expressed as linear chains, such as *Ubi-p5E*, are believed to be processed by DUBs down to mono-Ub (Larsen et al., 1998; Grou et al., 2015). Reduced levels of this specific Ub-encoding gene, but not of the other four, suggest that the presence of unanchored poly-Ub that is not conjugatable to other proteins could be perceived as *Ubi-p5E* product. What senses this unanchored chain and the processes through which *Ubi-p5E* is regulated are unclear. One might surmise that lower levels of Ubi-p5E translate into reduced mono-Ub protein in the fly and impaired Ub-dependent processes; in turn, this would be expected to lead to upregulation of other Ub-encoding genes or of DUBs that process it, none of which was detected by our analyses. Whether there is a physiological significance attached to the reduced levels of *Ubi-p5E* in the presence of Ub^6^-Stop presently is unclear; perhaps *Ubi-p5E* regulation can be utilized in the future to understand unanchored poly-Ub sensing at the protein level and its downstream events.

We did not detect coordinated changes in pathways that involve unanchored poly-Ub, such as NF-κB. One gene altered in both the Ub^6^-Stop and Ub^6^-GG conditions, *Takl1* (Tak1-like 1), is closely related to *Tak1*, a MAP3K required for the immune activation of NF-κB and JNK pathways (Emmerich et al., 2013; Silverman et al., 2003); little is known about fly *Takl1* itself. We caution here that the direction in the difference of transcript levels for *Takl1* differed between RNA-Seq and qRT-PCR results, arguing against a clear effect from unanchored poly-Ub on this gene's expression. Ub^6^-Stop also led to differential expression of two transcripts linked to immunity: the upregulation of *LysE* (Lysozyme E) and the downregulation of *DptA* (Diptericin A). LysE protein is expressed in the midgut, where it is believed to aid in the digestion of food-borne bacteria (Daffre et al., 1994). DptA is an antimicrobial protein whose expression is induced by the immune deficiency pathway via NF-κB-related proteins (Myllymaki et al., 2014; Tanji et al., 2007). While these genes are likely participants in immunity or some NF-κB processes, their limited number is not indicative of a major alteration in those processes, and our DAVID analyses did not point to an effect on immunity or NF-κB signaling as a whole.

The small number of enriched gene ontology terms and pathways that we observed is reasonable considering the limited input of differentially expressed genes, suggesting that the alteration of these genes does not overtly perturb critical physiological processes. One interesting exception is the enrichment of proteolysis and peptide catabolic process in both Ub^6^ species. While there is a consensus among the two types of untethered Ub chains in the upregulation of proteolysis-related genes, the number of impacted genes is not large. The GO category ‘proteolysis’ comprised about 15 genes, including two genes in the Trypsin family (*zetaTry* and *Jon65Aii*). Several proteolysis-related genes share homology with human PRSS genes (serine proteases; *CG18493*, *CG31266*, *CG8299*, *CG9763*, *CG11911*, *CG11912*, *CG6048*, *zetaTry*) and Aminopeptidase N (*CG31198*, *CG31343*, *CG42335*). Those genes might be involved with the disassembly of unanchored chains (Fig. 4), but it is unlikely that they act alone, as we observed previously that the proteasome is critically important for the degradation of unanchored chains in the fly (Blount et al., 2018). The increase in proteolysis-related genes suggests an attempt by the organism to remove these species via specific peptidases, which might indeed play a role in the removal of endogenous, unanchored poly-Ub; this possibility requires future attention. Our previous work showed that the proteasome is important for degradation of free poly-Ub (Blount et al., 2018). Perhaps the proteasome and these peptidases work in concert to dismantle free poly-Ub (Fig. 4).

A caveat to note is that our studies examined changes at the gene expression level. Perhaps control of untethered Ub^6^ species is coordinated by changes at the protein level. Changes in fly proteomics as a result of untethered poly-Ub await investigation. Suffice it to say here that, based on western blotting, none of the proteasome subunits we examined before showed a difference at the protein level (Blount et al., 2018). It is also important to note that the majority of the differentially expressed transcripts identified here are unannotated, leaving open the possibility that some of them have undiscovered roles in the handling of unanchored poly-Ub.

Because we utilized ubiquitous expression and whole flies for RNA-Seq analyses, it is possible that tissue- or system-specific responses are masked by conflicting changes in other tissues (Brown et al., 2014). We elected to examine changes in the whole fly, as done in prior work that identified numerous genes altered due to specific types of conditions or insults (Moskalev et al., 2014; Castillo et al., 2015; MacMillan et al., 2016; Jiang et al., 2017). As we continue to investigate unanchored poly-Ub *in vivo*, should we observe a need to examine transcriptome changes in a tissue-specific manner we will be well positioned to do so. However, our current work with unanchored poly-Ub species overall indicates that these members of the Ub family are not inherently toxic and do not present cells with particularly egregious insults that necessitate large, coordinated responses.

## MATERIALS AND METHODS

### Fly lines

Generation of transgenic *Drosophila* lines was described previously (Blount et al., 2018). Ubiquitous gene expression was driven by sqh-Gal4 (Brand et al., 1994; Brand and Perrimon, 1993; Kiehart et al., 2004; Franke et al., 2006, 2010; Todi et al., 2005, 2008), with all flies heterozygous for the transgene and the driver. In the case of controls, all flies were heterozygous for sqh-Gal4 on the genetic background of Ub^6^ flies. Crosses were maintained in diurnal incubators at 25°C and ∼60% humidity, on conventional cornmeal media. One-day-old adult offspring were collected for RNA isolation.

### RNA isolation

Total RNA was extracted from ten whole flies per group using TRIzol reagent (Invitrogen), following the manufacturer's protocol. RNA was then treated with TURBO DNase (Ambion) to eliminate contamination by DNA.

### RNA-Seq

RNA expression analysis was conducted at the Wayne State University Applied Genomics Technology Center. Four biological replicates were used for each genotype. An aliquot of the RNA was assessed by microfluidics using the ScreenTape for the Agilent 2200 TapeStation. The electrophoretogram, RNA integrity number (RIN), and the ratio of the 28S:18S RNA bands were collectively examined to determine overall quality of the RNA (Table S10). RNA-Seq, primed from the poly(A) tail, was used to determine expression profiles. Lexogen's QuantSeq 3′mRNA-Seq Library Prep Kit (FWD for Illumina) was utilized for building RNA-Seq libraries from 250 ng of total RNA in 5 µl of nuclease-free ultrapure water. Libraries were quantified on the Qubit and Agilent 2200 TapeStation using the DNA High Sensitivity Screen tape. The barcoded libraries were multiplexed at equimolar concentrations and sequenced with 50 bp reads in rapid mode on an Illumina HiSeq 2500. Data were de-multiplexed using Illumina's CASAVA 1.8.2 software. After quality was assessed (Andrews, 2010) reads were aligned to the *Drosophila* genome (Build dm3) with STAR_2.4 (Dobin et al., 2013) and tabulated for each gene region (Anders et al., 2015). Differential gene expression analysis was used to compare transcriptome changes between conditions using edgeR v.3.22.3 (Robinson et al., 2010). All conditions were individually compared (i.e. Ub^6^-GG versus control, Ub^6^-Stop versus control, and Ub^6^-Stop versus Ub^6^-GG) and transcripts were defined as significantly differentially expressed at absolute Log2 fold change >1, FDR <0.05. Dataset S1 contains all differentially expressed transcripts for each comparison.

### DAVID/Pathway analysis

Differentially expressed (absolute Log2 fold change >1, FDR<0.05) RNA-Seq transcripts at each level of comparison (Ub^6^-GG versus control, Ub^6^-Stop versus control, Ub^6^-Stop versus Ub^6^-GG) were used to identify affected pathways. Transcripts were separated into lists of upregulated and downregulated genes for each condition. Each list was uploaded into the Functional Annotation tool provided by DAVID (https://david.ncifcrf.gov, v. 6.8) as a gene list and submitted using the official gene symbol as identifier and *D. melanogaster* as background (or *Homo* *sapiens* where indicated). Charts were created from several terms of interest, including enriched Biological Process (BP_DIRECT), Molecular Function (MF_DIRECT), and Cellular Component (CC_DIRECT) gene ontology as well as KEGG Pathway (KEGG_PATHWAY) terms. Terms were included as enriched if *P*-value<0.05.

### Quantitative real time PCR

qRT-PCR was performed on select genes to validate RNA-Seq results. High-Capacity cDNA Reverse Transcription Kit (ABI) was used to obtain a cDNA library, following the manufacturer's protocol. Pre-amplification of the genes of interest was performed using TaqMan PreAmp Master Mix (Thermo Fisher Scientific). A Gilson 268 PIPETMAX liquid handling platform was used to plate Fast SYBR Green (ABI) qRT-PCR reactions in triplicate in 384-well plates. Messenger RNA levels were quantified with QuantStudio 5, using 2^−**ΔΔ**Ct^ (cycle threshold) methods and normalizing all transcripts to the reference gene, Rp49. All primer sequences are listed in Table S9.

## Supplementary Material

Supplementary information
