# Supplementary material for: Unanchored ubiquitin chains do not lead to marked alterations in gene expression in Drosophila melanogaster
Source: Biol Open. 2019 May 16;8(5):bio043372. doi: 10.1242/bio.043372 (PMC6550069; doi:10.1242/bio.043372)
Supplement: Supplementary information [file biolopen-8-043372-s1.pdf]

Table S1. Effects of Ub^6 expression throughout all *Drosophila* tissues and developmental stages, as previously published (Blount et al., 2018).

sqh-Gal4 driver (all tissues and stages)	Background Host Line	Ub^6-Stop	Ub^6-GG
**Expression**	None	Throughout development and throughout adulthood	Throughout development and throughout adulthood
**Protein detection**	No Ub^6	Highly robust signal by western blotting (various antibodies recognizing epitope tags or Ub) from simple lysates and from immunoprecipitations using whole flies and dissected tissues	Highly robust signal by western blotting (various antibodies recognizing epitope tags or Ub) from simple lysates and from immunoprecipitations from whole flies and dissected tissues
**Development**	Normal at all stages	Normal at all stages	Normal at all stages
**Eclosure of adults**	Normal	Normal; larval, pupal and pharate adult deaths not different from controls	Normal; larval, pupal and pharate adult deaths not different from controls
**Adult longevity**	Normal	Normal (not statistically significantly different from controls that contain driver on the background of the Ub^6 transgenes)	Normal (not statistically significantly different from controls that contain driver on the background of the Ub^6 transgenes)

Biology Open • Supplementary information

Table S2. Rationale for using DAVID analysis tool over IPA. Information was acquired through the databases themselves, as well as reviews written on the subject of functional analysis tools (Crona et al., 2015; Huang da et al., 2009b; Huang et al., 2007; Sherman et al., 2007; Gramates et al., 2017).

	DAVID	IPA
Pros	• *Drosophila*-specific gene analysis<br>• Groups functionally related genes and terms into manageable number of biological modules, helping to reduce redundancy<br>• Gene-to-term and term-to-gene analysis<br>• Improves "functional group" definition<br>• Fuzziness feature, that allows one gene to participate in more than one functional group<br>• Global view with "fuzzy heat map" visualization	• Places data in the context of public sources<br>• Canonical and overlapping pathways<br>• Generates plausible signaling cascades<br>• Builds interaction and regulatory networks<br>• Multiple profiling tools<br>• Pathway designer
Cons	• Running criteria must be fine tuned to avoid distortion<br>• "Orphan" terms, i.e. term exclusion because of undefined fit into a specific function<br>• Fewer designer tools	• Needs the most complete dataset on file<br>• Potential incongruence with GO terms<br>• Many of our genes of interest lack human orthologs<br>• Ignores our *Drosophila*-specific genes of interest

Biology Open • Supplementary information

Table S3. Expansion of Table 1. Symbols of differentially expressed genes organized by biological process. “CG” identifier denotes that the gene has not yet been named in *Drosophila.*

	Gene Ontology: Biological Process	
	**Term**	**Genes**
**Ub6-GG vs. Control**		
Upregulated	proteolysis	CG18493, CG31265, CG31266, CG4653, CG7025, CG8299, CG9673, ZetaTry
	peptide catabolic process	CG31198, CG31343, CG42335, CG8773
	mannose metabolic process	LManIII, LManV, LManVI
	protein deglycosylation	LManIII, LManV, LManVI
	melanin biosynthetic process from tyrosine	yellow-f2, Y
Downregulated	None detected	
**Ub6-Stop vs. Control**		
Upregulated	proteolysis	CG11911, CG11912, CG14820, CG31198, CG31343, CG42335, CG6048, CG7025, CG7631, CG9673, Jon65Aii, zetaTry
	peptide catabolic process	CG31198, CG31343, CG42335
	transmembrane transport	CG17751, CG32669, CG42825, CG4562, CG8785, MFS1
Downregulated	carbohydrate metabolic process	Mal-A1, Mal-A6, Mal-A7, Nmdmc
	folic acid-containing compound biosynthesis process	Nmdmc, pug
	tetrahydrofolate interconversion	CG3011, pug
	de novo' IMP biosynthetic process	AdSL, ade3
	one-carbon metabolic process	Nmdmc, pug
	oxidation-reduction process	CG12766, Cyp4p2, Nmdmc, pug
**Ub6-Stop vs. Ub6-GG**		
Upregulated	None detected	
Downregulated	carbohydrate metabolic process	Amy-d, Mal-A1, Mal-A4, Mal-A6, Mal-A7, Mal-A8, tobi
	oxidation-reduction process	CG12766, CG18003, CG1944, CG31075

Table S4. Expansion of Table 2. Symbols of differentially expressed genes organized by molecular function. "CG" identifier denotes that the gene has not yet been named in *Drosophila*.

	Gene Ontology: Molecular Function	
	**Term**	**Genes**
**Ub⁶-GG vs. Control**		
Upregulated	peptide binding	CG31198, CG31343, CG42335, CG8773
	metalloaminopeptidase activity	CG31198, CG31343, CG42335, CG8773
	alpha-mannosidase activity	LManIII, LManV, LManVI
	metallopeptidase activity	CG31198, CG31343, CG42335, CG8773
	serine-type endopeptidase activity	CG1304, CG31265, CG31266, CG4653, CG8299, CG9673, zetaTry
	carbohydrate binding	CG14499, LManIII, LManV, LManVI
	zinc ion binding	CG31198, CG31343, CG42335, CG7025, CG7631, CG8773, LManIII, LManV, LManVI
	hydrolase activity	CG14120, CG31266, vanin-like, CG4653, CG9673
	hydrolase activity, acting on C-N (not peptide) bonds, in linear amides	CG32751, vanin-like
**Ub⁶-Stop vs. Control**		
Upregulated	peptide binding	CG31198, CG31343, CG42335
	metalloaminopeptidase activity	CG31198, CG31343, CG42335
	serine-type endopeptidase activity	CG11911, CG11912, CG6048, CG9673, Jon65Aii, zetaTry
	metallopeptidase activity	CG31198, CG31343, CG42335
	hydrolase activity, acting on C-N (not peptide) bonds, in linear amides	CG32751, vanin-like
Downregulated	catalytic activity	CG5955, Mal-A1, Mal-A6, Mal-A7
	methenyltetrahydrofolate cyclohydrolase activity	Nmdmc, pug
	methylenetetrahydrofolate dehydrogenase (NADP+) activity	Nmdmc, pug
	formate-tetrahydrofolate ligase activity	Nmdmc, pug
	maltose alpha-glucosidase activity	Mal-A1, Mal-A7
	alpha-1,4-glucosidase activity	Mal-A1, Mal-A7
**Ub⁶-Stop vs. Ub⁶-GG**		
Upregulated	None detected	
Downregulated	maltose alpha-glucosidase activity	Mal-A1, Mal-A4, Mal-A7, Mal-A8, tobi
	alpha-1,4-glucosidase activity	Mal-A1, Mal-A4, Mal-A7, Mal-A8, tobi
	catalytic activity	Amy-d, Mal-A1, Mal-A4, Mal-A6, Mal-A7, Mal-A8

Table S5. Expansion of Table 3. Symbols of differentially expressed genes organized by KEGG pathway. "CG" identifier denotes that the gene has not yet been named in *Drosophila*.

	KEGG Pathway	
	**Pathway**	**Genes**
**Ub⁶-GG vs. Control**		
Upregulated	Lysosome	CG6903, LMANIII, LMANV, LMANVI, Vha100-4, Tsp29Fa
	Other glycan degradation	LMANIII, LMANV, LMANVI
Downregulated	None detected	
**Ub⁶-Stop vs. Control**		
Upregulated	None detected	
Downregulated	One carbon pool by folate	ade3, CG3011, Nmdmc, pug
	Metabolic pathways	ade, AdSL, CG11425, CG12766, CG13377, CG17224, CG30016, CG3011, CG6903, Mal-A1, Mal-A7, Nmdmc, pug, Ugt35b, vanin-like
	Galactose metabolism	CG12766, Mal-A1, Mal-A7
**Ub⁶-Stop vs. Ub⁶-GG**		
Upregulated	None detected	
Downregulated	Starch and sucrose metabolism	Amy-d, Mal-A1, Mal-A4, Mal-A7, Mal-A8, tobi
	Galactose metabolism	CG12766, Mal-A1, Mal-A4, Mal-A7, Mal-A8, tobi
	Metabolic pathways	Amy-d, CG11425, CG12766, CG18003, CG31075, Mal-A1, Mal-A4, Mal-A7, Mal-A8, tobi
	Glycerolipid metabolism	CG11425, CG12766, CG31075

Biology Open • Supplementary information

Table S6. Enriched Biological Process Gene Ontology terms as determined by DAVID analysis. Differentially expressed (Absolute Log2 Fold Change > 1, FDR < 0.05) RNA-Seq transcripts at each level of comparison (Ub^{6}-GG vs. Control, Ub^{6}-Stop vs. Control, Ub^{6}-Stop vs. Ub^{6}-GG) were assigned human orthologues by FlyBase.org and separated into lists of upregulated and downregulated genes for each condition. Each list was uploaded into DAVID's Functional Annotation tool (https://david.ncifcrf.gov, v. 6.8) as a gene list and submitted using the official gene symbol as identifier and *H. sapiens* as background. The term BP_DIRECT was selected for chart creation within the Gene Ontology category, and terms were included as enriched if p-value < 0.05. * indicates overlap in terms between Ub^{6}-GG vs. Control and Ub^{6}-Stop vs. Control comparisons.

	**Gene Ontology: Biological Process**			
	**Term**	**# of genes**	**p-value**	**Genes**
**Ub^{6}-GG vs. Control**				
Upregulated	proteolysis *	9	2.30E-05	ANPEP, CPB1, CFD, HPN, KLK9, MEP1A, PRSS16, PRSS36, TPSD1
	termination of signal transduction	2	7.30E-03	GBA, SMPD1
	response to thyroid hormone	2	1.50E-02	GBA, HPN
	response to pH	2	2.90E-02	GBA, TTPA
	regulation of cell shape	3	4.60E-02	BAIAP2, FMNL1, HPN
Downregulated	None detected			
**Ub^{6}-Stop vs. Control**				
Upregulated	proteolysis *	7	7.20E-04	ANPEP, AZU1, CPA3, CPB1, HPN, MEP1A, TPSD1
	pyrimidine nucleoside salvage	2	2.60E-02	CDA, UPP1
	biotin metabolic process	2	3.00E-02	BTD, SLC5A6
Downregulated	folic acid metabolic process	3	1.30E-04	MTHFD1, MTHFD2, SHMT1
	purine nucleobase biosynthetic process	2	4.80E-03	GART, SHMT1
	de novo IMP biosynthetic process	2	5.70E-03	AdSL, GART
	folic acid-containing compound biosynthesis process	2	5.70E-03	MTHFD2, MTHFD1
	glycine metabolic process	2	8.50E-03	GART, SHMT1
	tetrahydrofolate interconversion	2	9.50E-03	MTHFD1, SHMT1
	tetrahydrofolate metabolic process	2	9.50E-03	MTHFD2, SHMT1
	purine nucleotide biosynthetic process	2	1.10E-02	ADSL, MTHFD1
	purine ribonucleoside monophosphate biosynthetic process	2	1.20E-02	ADSL, GART
	oxidation-reduction process	4	1.70E-02	AKR1A1, CYP4B1, MTHFD1, MTHFD2
	one-carbon metabolic process	2	2.80E-02	MTHFD1, MTHFD2
	amino acid transport	2	3.30E-02	SLC3A1, SLC36A1
	protein tetramerization	2	3.70E-02	ADSL, SHMT1
**Ub^{6}-Stop vs. Ub^{6}-GG**				
Upregulated	None detected			
Downregulated	carbohydrate metabolic process	3	2.20E-03	ALDH2, AMY2B, SLC3A1

Biology Open • Supplementary information

Table S7. Enriched Molecular Function Gene Ontology terms as determined by DAVID analysis. Differentially expressed (Absolute Log2 Fold Change > 1, FDR < 0.05) RNA-Seq transcripts at each level of comparison (Ub^{6}-GG vs. Control, Ub^{6}-Stop vs. Control, Ub^{6}-Stop vs. Ub^{6}-GG) were assigned human orthologues by FlyBase.org and separated into lists of upregulated and downregulated genes for each condition. Each list was uploaded into DAVID's Functional Annotation tool (https://david.ncifcrf.gov, v. 6.8) as a gene list and submitted using the official gene symbol as identifier and *H. sapiens* as background. The term MF_DIRECT was selected for chart creation within the Gene Ontology category, and terms were included as enriched if p-value < 0.05. * indicates overlap in terms between Ub^{6}-GG vs. Control and Ub^{6}-Stop vs. Control comparisons.

	Gene Ontology: Molecular Function			
	**Term**	**# of genes**	**p-value**	**Genes**
**Ub^{6}-GG vs. Control**				
Upregulated	serine-type peptidase activity	4	4.80E-04	CFD, HPN, PRSS16, TPSD1
	serine-type endopeptidase activity *	5	3.30E-03	CFD, HPN, KLK9, PRSS36
	zinc ion binding *	8	2.10E-02	TRAIP, ANPEP, CPB1, CDA, ENPEP, MAN2B1, MEP1A, SMPD1
	apolipoprotein binding	2	3.30E-02	CANX, CSARB1
	mannose binding	2	4.70E-02	CD207, MAN2B1
Downregulated	None detected			
**Ub^{6}-Stop vs. Control**				
Upregulated	zinc ion binding *	8	1.20E-02	TRAIP, ANPEP, BNC2, CPA3, CPB1, CDA, MEP1A
	serine-type endopeptidase activity *	4	1.80E-02	AZU1, HPN, TFPI, TPSD1
Downregulated	methylenetetrahydrofolate dehydrogenase (NAD+) activity	2	2.70E-03	MTHFD1, MTHFD2
	methylenetetrahydrofolate dehydrogenase (NADP+) activity	2	3.50E-03	MTHFD1, MTHFD2
	methenyltetrahydrofolate cyclohydrolase activity	2	3.50E-03	MTHFD1, MTHFD2
	formate-tetrahydrofolate ligase activity	2	3.50E-03	MTHFD1, MTHFD2
	catalytic activity	3	1.20E-02	ADSL, GART, SLC3A1
	amino acid transmembrane transporter activity	2	4.10E-02	SLC3A1, SLC36A1
**Ub^{6}-Stop vs. Ub^{6}-GG**				
Upregulated	None detected			
Downregulated	oxidoreductase activity	3	2.00E-03	ALDH2, AKR1A1, HAO2
	electron carrier activity	2	3.20E-02	ALDH2, AKR1A1

Table S8. Enriched KEGG Pathway terms as determined by DAVID analysis. Differentially expressed (Absolute Log2 Fold Change > 1, FDR < 0.05) RNA-Seq transcripts at each level of comparison (Ub^{6}-GG vs. Control, Ub^{6}-Stop vs. Control, Ub^{6}-Stop vs. Ub^{6}-GG) were assigned human orthologues by FlyBase.org and separated into lists of upregulated and downregulated genes for each condition. Each list was uploaded into DAVID's Functional Annotation tool (https://david.ncifcrf.gov, v. 6.8) as a gene list and submitted using the official gene symbol as identifier and *H. sapiens* as background. The term KEGG_PATHWAY was selected for chart creation within the Pathway category, and terms were included as enriched if p-value < 0.05. + indicates overlap in terms between Ub^{6}-Stop vs. Control and Ub^{6}-Stop vs. Ub^{6}-GG comparisons, ^ indicates overlap in terms across all comparisons.

	KEGG Pathway			
	**Pathway**	**# of genes**	**p-value**	**Genes**
**Ub^{6}-GG vs .Control**				
Upregulated	Lysosome	5	1.00E-03	ATP6V0A4, CD63, GBA, MAN2B1, SMPD1
	Metabolic pathways ^	10	2.90E-02	ALPPL2, ANPEP, ATP6V0A1, BTD, CDA, CHDH, CYP3A4, DPM3, GBA, SMPD1
Downregulated	None detected			
**Ub^{6}-Stop vs. Control**				
Upregulated	Drug metabolism- other enzymes	3	7.00E-03	CDA, UGT2A3, UPP1
	Protein digestion and absorption	3	2.40E-02	CPA3, CPB1, MEP1A
Downregulated	One carbon pool by folate	4	4.50E-06	MTHFD2, MTHFD1, GART, SHMT1
	Metabolic pathways ^	9	2.40E-04	ADSL, AKR1A1, CYP4F2, GART, HGSNAT, MTHFD1, MTHFD2, PLPP1, SHMT1
	Biosynthesis of antibiotics +	5	3.60E-04	TGDS, ADSL, AKR1A1, GART, SHMT1
	Glycerolipid metabolism +	3	4.40E-03	AKR1A1, LPL, PLPP1
**Ub^{6}-Stop vs. Ub^{6}-GG**				
Upregulated	None detected			
Downregulated	Metabolic pathways ^	6	8.90E-04	ALDH2, AKR1A1, AMY2B, CYP4F2, HAO2, PLPP1
	Glycerolipid metabolism +	3	1.00E-03	ALDH2, AKR1A1, PLPP1
	Biosynthesis of antibiotics +	3	1.30E-02	ALDH2, AKR1A1, HAO2

Biology Open • Supplementary information

Table S9. All primer sequences used for qRT-PCR and their corresponding genes' expression patterns in *Drosophila melanogaster*, according to FlyAtlas.org. "CG" identifier denotes that the gene has not yet been named in *Drosophila*.

Gene Target	Forward primer 5′-3′	Reverse primer 5′-3′	Localization
**Rp49**	AGATCGTGAAGAAGCGCACCAAG	CACCAGGAACTTCTTGAATCCGG	Global
**CG11911**	GCCAACATGCCTTCCATTTAC	GCGATTTCGATCCTGGCTAATA	Midgut
**CG1304**	TCCACGAGGAGTATGGTAACT	GGTAGATCGATGGGCTGAATAC	Midgut
**CG2650**	GATGTCCAGCCGAAGATCAA	GAAGAACTCGTCGTAGGGAATG	Rectal pad and eye
**CG32751**	CGTATTCACCTGCTCTGGAAA	CTGCGGATATGTGACCCTAATC	Midgut and Spermatheca
**CG4653**	GGTGGTCCTGAATGCGAATA	TGGAGGACATGACTGAGAGA	Midgut, hindgut, and Malpighian tubules
**CG7631**	CGTATTCACCTGCTCTGGAAA	CTGCGGATATGTGACCCTAATC	Midgut
**Drsl3**	GGTGCAGATGATATTCCTGTTTG	GACCTCCGAAAGTTCCAGATAG	Midgut
**LysE**	GCTCGTTGGGCCTGTATT	GAAGATTCCGTAGTCGTTGGAG	Midgut and accessory glands
**Mal-A7**	GGCAAACGAATTGGACCTAAAG	CGTAGCCCTTCTCTCGATTTAC	Midgut
**qin**	CTGATCGCCAGACAAGTACAA	GACCGTAGACCGTCAAGTAATC	Testis, ovary, and eye
**Takl1**	TCCCTCCACAACTACCTCTATG	AGGCGATCTTGCACTTTACC	Midgut, hindut, and Malpighian tubules
**Ubi-p5E**	GGACGTCCGAGCAAGTAAAA	ATGGCTCAACCTCCAAAGTG	Global

Biology Open • Supplementary information

Table S10. Overall RNA quality. Before RNA-Seq, an aliquot of the RNA was assessed by microfluidics using the ScreenTape for the Agilent 2200 TapeStation. The electrophoretogram (not pictured), RNA Integrity Number (RIN), and the ratio of the 28S:18S RNA bands are collectively examined to determine overall quality of the RNA.

Sample Name	RINe	28S/18S (Area)	Conc. (ng/uL)
GG-1	9.3	0.1	125
GG-2	7.6	N/A	37.4
GG-3	8.6	0.1	111
GG-4	8.9	0.1	95.3
Stop-1	6.9	N/A	42.2
Stop-2	6.2	N/A	41.4
Stop-3	8.4	0.2	141
Stop-4	8.4	0.2	167
Ctrl-1	6.0	0.2	76.3
Ctrl-2	7.5	0.3	199
Ctrl-3	8.0	0.1	65.1
Ctrl-4	7.8	0.2	101

Dataset S1. List of all differentially expressed genes and their human orthologues. Only the top scoring orthologue predictions from FlyBase.org are listed.

Click here to download dataset S1
